# Cecal Schwannoma: A Rare Cause of Gastrointestinal Bleeding in a Young Woman with Review of Literature

**DOI:** 10.1155/2011/142781

**Published:** 2011-06-15

**Authors:** Kalyan Kanneganti, Harish Patel, Masooma Niazi, Kavitha Kumbum, Bhavna Balar

**Affiliations:** ^1^Division of Gastroenterology, Department of Medicine, Bronx-Lebanon Hospital Center, 1650 Grand Concourse, Albert Einstein College of Medicine, Bronx, 10457, USA; ^2^Department of Medicine, Bronx-Lebanon Hospital Center, 1650 Grand Concourse, Albert Einstein College of Medicine, Bronx, New York 10457, USA; ^3^Department of Pathology, Bronx-Lebanon Hospital Center, 1650 Grand Concourse, Albert Einstein College of Medicine, Bronx, New York 10457, USA

## Abstract

Schwannomas are rare mesenchymal tumors of the gastrointestinal tract. Occurrence of these tumors is more common in the stomach than in the large intestine. These tumors usually present as polypoidal intraluminal lesions and based on their location can present with rectal bleeding, colonic obstruction, and abdominal pain or defecation disorders. We present a case of a thirty-five-year-old woman who presented with abdominal pain and melena. Patient was diagnosed with a nonobstructing superficially ulcerated mass in the cecum on colonoscopy and required right hemicolectomy. A very rare pathological diagnosis of cecal schwannoma was made postoperatively.

## 1. Introduction

Schwannomas originate from the Schwann cells, which form the neural sheath and encompass the nerves of the myenteric plexus. Schwannomas were first described by Verocay in 1910. The exact incidence of schwannomas is not known. Review of the literature suggests that soft tissue tumors contribute about 1% of all gastrointestinal tumors [1]; and schwannomas account for about 2% to 8% of all gastrointestinal mesenchymal tumors [1, 2]. This is a case of a young woman who presented with melena and colonoscopy showed a mass in the cecum requiring surgery revealing a very rare schwannoma.

## 2. Case Report

**Figure 1:**
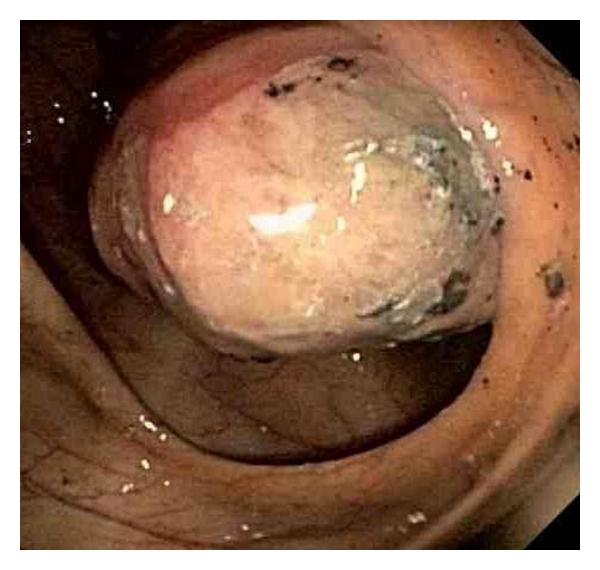
Superficially ulcerated cecal mass.

**Figure 2:**
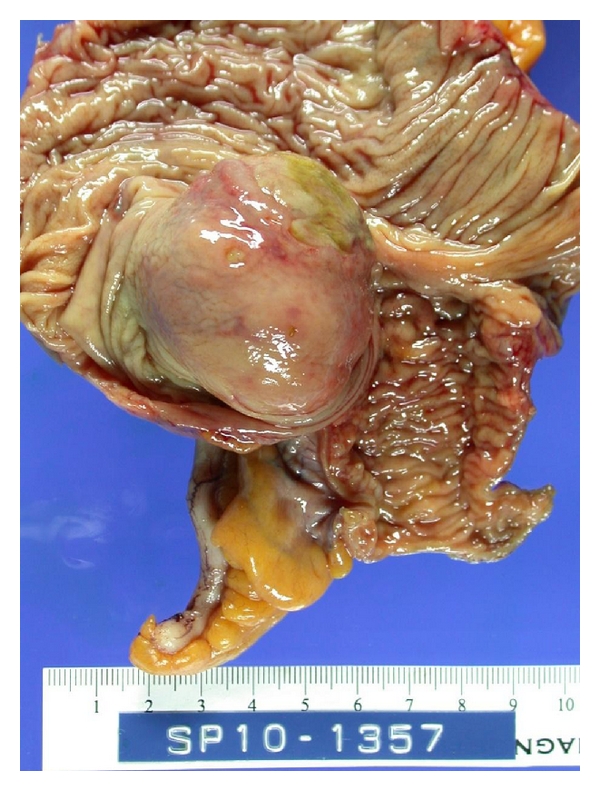
Right hemicolectomy specimen with polypoidal cecal mass.

**Figure 3:**
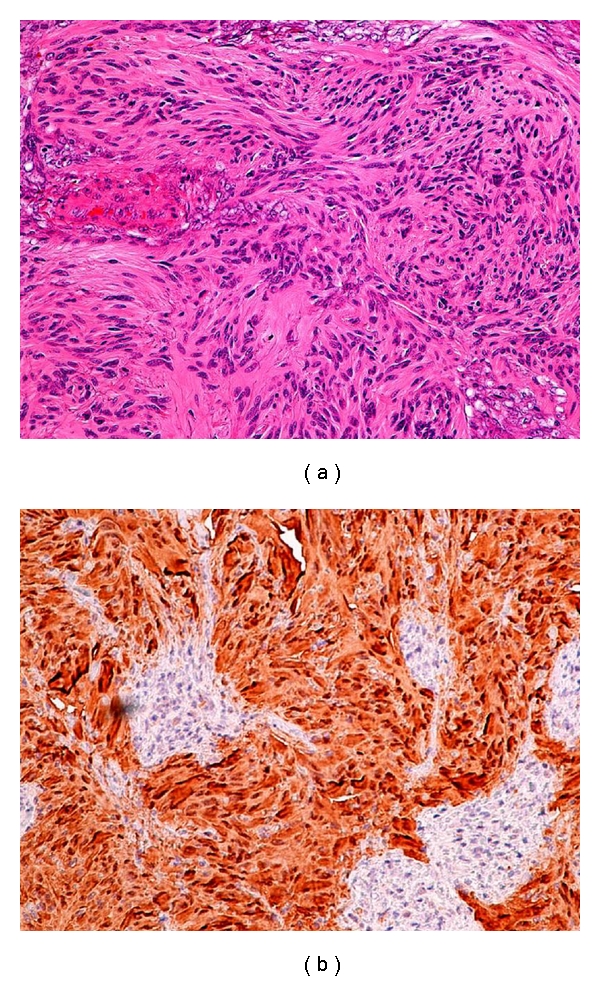
(a) The tumor cells arranged in fascicular and nodular pattern. (b) S-100 Protein [+]. The tumor cells illustrate strong immunoreactivity to S-100 protein.

A 35-year-old woman presented to our emergency department with epigastric pain and melena for two weeks. Abdominal pain was described as sharp in character, severe in intensity, and worse after eating food. Patient also reported nausea, vomiting, and some red blood in the stool for two days. Medical history included gastroesophageal reflux disease, herniated disk, migraine headache and depression. Prior medications included nonsteroidal anti-inflammatory drugs. Physical examination was unremarkable except for epigastric tenderness. Laboratory data showed hemoglobin of 9.2 g/d with iron deficiency. Nasogastric lavage did not reveal any active bleeding. With an initial impression of possible upper gastrointestinal lesion an upper gastrointestinal endoscopy was performed. It did not reveal any pathology to explain etiology of melena. A colonoscopy performed revealed a nonobstructing superficially ulcerated mass in the cecum (Figure 1). Biopsies of this mass only revealed an ulceration of the colonic mucosa without any evidence of malignant cells. A computerized tomography scan of the abdomen identified a 4 × 4 × 3.5 cm mass in the cecum. Surgical consultation was obtained. After discussion with the patient and her family a right hemicolectomy was performed. Operative findings revealed a large cecal mass, mesenteric lymphadenopathy, and normal liver without evidence of any peritoneal metastasis. Gross examination of the right hemicolectomy specimen showed a polypoid, well-circumscribed intraluminal firm cecal mass, measuring 4.2 × 4 cm. The cut surface was homogenous tan to yellow with no necrosis or hemorrhage (Figure 2). Microscopically, the tumor was comprised of cellular spindle cells arranged in fascicular and nodular pattern. The nuclei are ovoid with pointy ends. No increased mitotic activity or necrosis was seen (Figure 3(a)). The tumor was surrounded by a prominent lymphoid cuffing. On immunostains the tumor cells were strongly and diffusely positive for S-100 protein (Figure 3(b)) and negative for CD34, SMA, Desmin, C-Kit [CD: 117], and DOG-1. A pathological diagnosis of cecal schwannoma was made. Postsurgical recovery was uneventful. Subsequent followup in the clinic was unremarkable for any new gastrointestinal symptoms.

## 3. Discussion

Schwannomas of the gastrointestinal tract are being increasingly diagnosed with the advent of newer immunohistochemical staining. Schwannomas when present in the gastrointestinal tract are common in the stomach and are rarely found in the large intestine [3]. In a review of 20 cases by Miettinen et al. of the files of the Armed Forces Institute of Pathology, colorectal schwannomas were more frequently present in the cecum compared to the rectosigmoid region. In comparison, a review of the Japanese literature in 46 cases by Inagawa et al. reported rectum as the commonest location for these tumors [4]. Schwannomas of the colon are distributed equally among males and females. They generally tend to appear after the sixth decade of the life [5]. The tumors usually manifest themselves as intraluminal polyps and can present with gastrointestinal bleeding from ulceration, colonic obstruction, or abdominal pain [6]. 

Schwannomas tend to share the gross morphological features with the other submucosal tumors and may be hard, solid, ulcerated, or calcified in rare cases [4, 7]. It is often difficult to biopsy them because of their hardness [7]. Preoperative endoscopy guided biopsy has a poor yield and may not contribute in the diagnosis of the colonic schwannoma though EUS guided biopsy may increase the yield [7–9]. In a review of 41 cases of schwannomas of the large intestine in Japan done by Tomozawa et al., only 10% of cases were identified pre-operatively [7]. Despite all efforts done preoperatively the differentiation of these tumors from other malignant stromal tumors, is mostly possible after histological examination of the resected specimen. Computerized scans, barium enemas, and magnetic resonance imaging may reveal these tumors as encapsulated masses arising from the colon mucosa, but there is still a lack of set criteria to differentiate benign from the malignant stromal tumors [10]. Though on computerized scans, their homogeneous attenuation pattern may help differentiate from gastrointestinal stromal tumors [11]. Colonic schwannomas have intense avidity for flourodeoxy D-glucose (FDG) similar to the other malignant gastrointestinal stromal tumors hence are indistinguishable on Positron emission tomography [6]. In accessible sites endosonographic features of irregular extraluminal margins, cystic spaces, and lymph node with malignant pattern help differentiate benign stromal cell tumors from the malignant tumors [12]. 

Histopathological and immunohistochemical study of the tumor cells remains the most important diagnostic tool. In an analysis done by Miettinen et al. [13], the commonest location for schwannomas in the large bowel is the cecum and spindle cells with a trabecular pattern and vague or no verocay bodies was the most frequently encountered histological variant. A lymphoid cuff with germinal centers typically surrounds these tumors. Schwannoma cells are immune reactive to S-100 protein, vimentin and negative to Smooth Muscle Actin (SMA) and CD117 (KIT). Smooth muscle tumors have desmin and SMA reactivity. Gastrointestinal Stromal Tumor (GIST) with KIT mutation/expression show immunopositivity to CD117 and CD34 [14, 15]. A definitive diagnosis of Schwannoma is rarely made preoperatively as it is difficult to obtain a large biopsy specimen to make a pathological diagnosis [4]. The tumors are generally considered benign and surgical removal of the tumor with the free margins remains the first line of the treatment. In locally advanced tumors the surgical management should be combined with vigilant postoperative followup. However because of the lack of specific histological grading for this tumor all patients need a close followup. Most cases undergo aggressive surgical intervention because of lack of the tissue diagnosis preoperatively.

## 4. Conclusion

Schwannomas of colon are rare tumors of the gastrointestinal tract and classified under mesenchymal tumors. They manifest themselves with varied clinical features depending on their location in the gastrointestinal tract. Endoscopic biopsy at times may fail to achieve an appropriate tissue diagnosis as in our case, though at accessible sites endoscopic ultrasound guided fine needle aspiration may increase the yield. Management differs for various soft tissue tumors and hence it is vital to distinguish them based on their immunehistochemistry. Schwannoma of colon needs excision with tumor-free margins without chemo-or radiation therapy. Our case illustrates an example of a very rare tumor presenting with melena in a young patient requiring surgery.
